# Polymorphic variants (p.Ser141Ser and p.Arg737Gly) at the *NAGLU* gene are really indicative of pseudodeficiency alleles?

**DOI:** 10.1186/s13052-019-0657-3

**Published:** 2019-05-14

**Authors:** Diana Rojas Malaga, Sandra Leistner-Segal, Ana Carolina Brusius-Facchin

**Affiliations:** 1 Medical Genetics Service, Hospital de Clínicas de Porto Alegre (HCPA), Rua Ramiro Barcelos 2350, Porto Alegre, RS 90035-903 Brazil; 2 Postgraduate Program of Genetics and Molecular Biology, Universidade Federal do Rio Grande do Sul (UFRGS), Av. Bento Gonçalves 9500, Porto Alegre, RS 91501-970 Brazil; 3 Postgraduate Program in Medicine: Medical Science, Universidade Federal do Rio Grande do Sul (UFRGS), Rua Ramiro Barcelos 2400, Porto Alegre, RS 90035-003 Brazil

**Keywords:** *NAGLU* gene, Alpha-N-acetyl-D-glucosaminidase, Pseudodeficiency allele, Sanfilippo B syndrome

## Abstract

Filocamo et al. recently published a paper describing the presence of a pseudodeficiency allele, constituted by p.Ser141Ser and p.Arg737Gly polymorphisms at the *NAGLU* gene, which leads to a reduced level of the alpha-N-acetyl-D-glucosaminidase activity. Based on analysis performed in Brazilian patients, using a customized gene panel containing *SGSH*, *NAGLU*, *HGSNAT* and *GNS* we observed that p.Ser141Ser (rs659497) and p.Arg737Gly (rs86312) variants were present in homozygosis in all of our MPS IIIB patients and in the majority of MPS IIIA, IIIC and IIID patients, and there was no significant decrease of the alpha-N-acetyl-D-glucosaminidase enzyme activity in this group when compared with those without the “pseudodeficiency allele”. Thus, we suggest that these two variants are not producing a pseudodeficiency allele.

To the Editor

We read with great interest the article by Filocamo et al. [1] in the November 2018 issue of Italian Journal of Pediatrics. In this manuscript, the authors mention that a complication for molecular analysis of *NAGLU* gene (associated with Sanfilippo B syndrome or also called mucopolysaccharidoses type IIIB) is the presence of a pseudodeficiency allele, constituted by p.Ser141Ser and p.Arg737Gly polymorphisms simultaneously, which leads to a reduced level of the alpha-N-acetyl-D-iglucosamnidase activity.

We have been working on the diagnosis of mucopolysaccharidoses (MPS) over the past 35 years, and during this time, MPS diagnosis was confirmed in 1184 Brazilian patients, of which, 192 were affected by one of the four subtypes of MPS III [2]. As part of our MPS III investigation, after detection of heparan sulphate glycosaminoglycan in urine, we performed different enzyme activity assays in leukocytes in order to reach the definitive diagnosis and to determine the MPS III subtype [2]. Following this step, approximately 40% of our MPS III patients, were analyzed by targeted-next generation sequencing (NGS), using a customized gene panel containing *SGSH*, *NAGLU*, *HGSNAT* and *GNS* which was designed to simultaneous sequence the entire coding regions plus 20pb of intron-exon junction of these genes. Pathogenic variants were detected in all the cases. Therefore, we have extensive biochemical and molecular data of our group of patients.

Based in our data, p.Ser141Ser (rs659497) and p.Arg737Gly (rs86312) variants were present in homozygosis in all our MPS IIIB patients analyzed by NGS. For these patients, the enzyme activities were below the reference range, as they are clinically affected by MPS IIIB as proven by mutation analysis.

On the other hand, we have patients with other types of MPS III (IIIA, IIIC and IIID) where the same alterations that are said to cause the pseudodeficiency allele were present in homozygosis. These variants were detected because patients had clinical phenotype of MPS III and were analyzed in the same NGS panel used for the 4 types of MPS III. For those, the NAGLU enzyme activity was within the normal range when compared to normal controls as shown in Table 1.

**Table 1:** Genotype and alpha-N-acetyl-D-glucosaminidase activity for MPS III patients

	n	p.Ser141Ser (c.423 T > C)	p.Arg737Gly (c.2209C > G)	alpha-N-acetyl-D-glucosaminidase activity in leukocytes (reference value: 10–34 nmol/17 h/mg protein)
MPS IIIB patients	12	C/C	G/G	0,25 ± 0,17
Other MPS III patients	18	C/C	G/G	14,5 ± 3,5
	2	C/C	C/G	19,8 ± 3,2
	2	T/C	C/G	16,5 ± 1,5

These two variants were also present in homozygosis in one MPSIIIA patient from Turkey and two MPS IIIB from Ecuador.

For our group of MPS III patients, allele frequencies of p.Ser141Ser and p.Ar737Gly were 0.9706 and 0.9412, respectively. In addition, Exome Aggregation Consortium (ExAC) reported that frequency for p.Ser141Ser is 0.9947 and 0.9055 for p.Arg737Gly [3]. Furthermore a Brazilian mutation database (ABraOM), which comprised the exomic variants of a cohort of 609 healthy Brazilian elderly, reported 602 homozygotes for p.Ser141Ser (0.9942) and 459 homozygotes for p.Arg737Gly (0.8686) [4].

Pseudodeficiency alleles are not rare in Lysosomal Disorders, including several MPS disorders [5–10], nevertheless the high frequency of both polymorphisms in worldwide populations and the fact that the presence of these alleles does not alter the MPS IIIB enzyme activity when present, suggests that these two variants are not producing a pseudodeficiency allele. Thus, no false positive results and no alteration on the protein product or changes in gene expression are detected in the presence of these two variants.

## Abbreviations

ExAC: Exome Aggregation Consortium
*GNS*: N-acetylglucosamine-6-sulfatase
*HGSNAT*: Heparan-Alpha-Glucosaminide N-Acetyltransferase
MPS: Mucopolysaccharidoses
*NAGLU*: Alpha-N-acetyl-D-glucosaminidase
NGS: Next Generation Sequencing
*SGSH*: N-sulphoglucosamine sulphohydrolase
